# Circulating Antinephrin Antibodies in Adult Chinese Patients With IgAN and Nephrotic-Range Proteinuria

**DOI:** 10.1016/j.ekir.2026.106640

**Published:** 2026-06-02

**Authors:** Fenglei Si, Yue Shu, Chen Tang, Jing Huang, Pei Chen, Jicheng Lv, Li Zhu, Sufang Shi, Xujie Zhou, Hong Zhang, Minghui Zhao, Zhao Cui, Lijun Liu

**Affiliations:** 1 Renal Division, Peking University First Hospital, Peking University Institute of Nephrology, Beijing, China; 2 Key Laboratory of Renal Disease, Ministry of Health of China, Beijing, China; 3 Key Laboratory of Chronic Kidney Disease Prevention and Treatment (Peking University), Ministry of Education, Beijing, China; 4 Research Units of Diagnosis and Treatment of Immune-Mediated Kidney Diseases, Chinese Academy of Medical Sciences, Beijing, China

**Keywords:** IgA nephropathy, minimal change disease, nephrin, nephrotic syndrome, podocytopathy

## Abstract

**Introduction:**

Antinephrin autoantibodies are detectable in podocytopathies such as minimal change disease (MCD) and primary focal segmental glomerulosclerosis (FSGS); however, their prevalence and clinical relevance in IgA nephropathy (IgAN) with nephrotic-range proteinuria (NRP) and nephrotic syndrome (NS) are unclear.

**Methods:**

This single-center retrospective study enrolled 234 adults with biopsy-proven IgAN and NRP (≥ 3.5 g/d) from 2003 to 2020. Plasma antinephrin IgG and IgM were measured using enzyme-linked immunosorbent assay. Prespecified subgroups included NS (*n* = 105) and concurrent MCD (*n* = 17). Clinicopathologic features, treatment, and outcomes were compared by serostatus, with MCD and FSGS cohorts as references.

**Results:**

Overall antinephrin seropositivity was 8% (IgG: 6%, IgM: 2%). The seropositivity rate was 35% in patients with MCD versus 6% in those without. In patients with NS, seropositivity was 14%; rates were 35% with and 10% without MCD. Seropositive patients had heavier proteinuria (median: 8.1 vs. 4.9 g/d), lower serum albumin (21.9 ± 6.5 vs. 31.0 ± 7.2 g/l), fewer urinary red blood cells (RBCs), and similar estimated glomerular filtration rate (eGFR). Their biopsies showed milder chronic lesions (more M0/S0/T0). Seropositive patients more often received glucocorticoids and achieved higher complete remission (CR) rates (67% vs. 30% overall; 80% vs. 43% within NS) and fewer composite renal endpoints (0% vs. 28%). Multivariable analysis showed that antinephrin positivity was associated with a trend toward higher CR rates.

**Conclusion:**

Circulating antinephrin antibodies mark a podocytopathy-like phenotype in IgAN with MCD (MCD-IgAN) and define a distinct NS phenotype in patients with IgAN without overt podocytopathy. Targeted serologic testing could facilitate subgroup identification and guide tailored management.

## Graphical abstract

**Figure:**
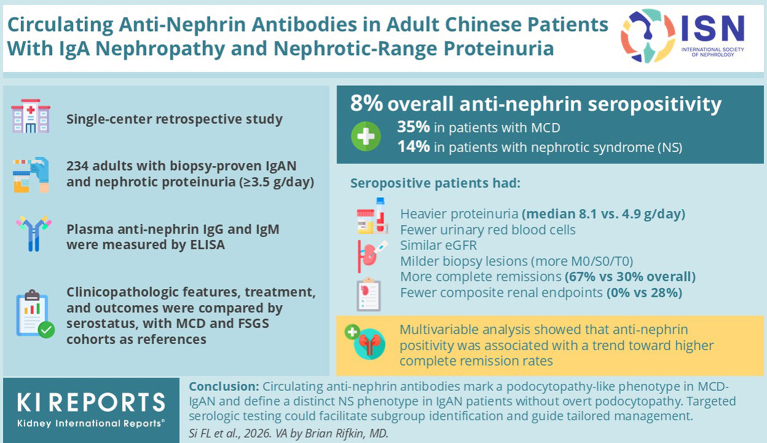

IgAN, the most common primary glomerulonephritis worldwide, progresses to end-stage kidney disease in approximately 30% to 40% of patients within 20 to 30 years of diagnosis.^1^^,^^2^ IgAN typically presents with recurrent macrohematuria episodes, persistent microhematuria, and proteinuria. NS is an uncommon presentation, reported in only 5% to 15% of patients with IgAN.^3^^,^^4^

The occurrence of NRP or NS usually indicates widespread disruption of the glomerular filtration barrier. Historically, such presentations have been presumed to reflect superimposed MCD and managed accordingly in patients with IgAN.^5^^,^^6^ However, accumulating evidence indicates that nephrotic IgAN is heterogeneous, not uniformly MCD-driven, and its immunopathogenesis remains incompletely understood. Of note, current Kidney Disease: Improving Global Outcomes guidelines emphasize that IgAN with NRP and IgAN with NS (IgAN-NS) do not reflect the same underlying disease spectrum or treatment approach. NRP without NS is often associated with secondary FSGS and substantial chronicity, whereas NS is more suggestive of concomitant podocytopathy.

Podocyte injury is central to the genesis of proteinuria. Nephrin, a key transmembrane component of the slit diaphragm, maintains podocyte structural integrity and intracellular signaling.^7^ Recent studies have identified circulating ant-nephrin autoantibodies in patients with MCD or primary FSGS, with reported positivity rates of approximately 30% to 90%, supporting the concept that a subset of these disorders constitutes an autoimmune podocytopathy.^8^^,^^9^

Against this background, assessing antinephrin antibodies in patients with IgAN presenting with NRP or NS may help delineate a putative immune podocytopathy subtype and refine risk stratification and management. Specifically, we hypothesized that antinephrin antibodies could serve as a diagnostic adjunct to identify patients with IgAN with concurrent MCD or podocytopathic features, a predictive biomarker for favorable steroid response, a prognostic indicator for renal outcomes, and a phenotypic marker of podocyte injury in this heterogeneous population. Accordingly, we quantified circulating antinephrin antibodies in a single-center cohort of IgAN with NRP or NS and evaluated their associations with clinicopathologic characteristics and treatment outcomes.

## Methods

### Study Design and Patient Enrollment

We conducted a single-center, consecutive retrospective cohort study including 1995 patients with biopsy-proven IgAN diagnosed between 2003 and 2020 at Peking University First Hospital. For the present analysis, we identified 318 patients with NRP, defined as 24-hour urinary total protein ≥ 3.5 g/d. We excluded 17 patients with secondary IgAN (e.g., IgA vasculitis [Henoch-Schönlein purpura], systemic lupus erythematosus, or cirrhosis), 6 with coexisting diabetic nephropathy, 6 with baseline eGFR ≤ 15 ml/min per 1.73 m^2^, 6 with missing prespecified follow-up variables, and 49 without a blood sample at the time of kidney biopsy, yielding a final analytic cohort of 234 patients, of whom 105 had NS and 17 had biopsy-proven MCD. MCD-IgAN was defined as mild mesangial proliferative IgAN on light microscopy, mesangial IgA deposits on immunofluorescence, and extensive podocyte foot process effacement (> 75%) with mesangial electron-dense deposits on electron microscopy. This study received approval from the Ethics Committee at the Peking University First Hospital [Ethics Approval Number: 2013 (548)], and all participating patients provided informed consent.

### Data Collection

Clinical parameters were retrospectively abstracted at baseline (time of biopsy) and during follow-up, including age at biopsy, gender, body mass index, mean arterial pressure, serum albumin, serum creatinine, eGFR, 24-hour urinary total protein, and microscopic hematuria; eGFR was calculated using the Chronic Kidney Disease Epidemiology Collaboration equation.^10^ Renal biopsy specimens were evaluated using direct immunofluorescence, light microscopy, and electron microscopy. Slides were reviewed independently, in a blinded fashion, by 2 renal pathologists and scored according to the Oxford classification.^11^ Any diagnostic discrepancies between the 2 pathologists were resolved through a consensus review of the biopsies.

Treatment regimens were retrospectively abstracted from medical records, including corticosteroids; other immunosuppressive agents (cyclophosphamide, leflunomide, mycophenolate mofetil, and calcineurin inhibitors); and renin-angiotensin-aldosterone system inhibitors. Treatment responsiveness was assessed according to prespecified criteria, and both follow-up duration and attainment of the prespecified endpoints were recorded.

### Detection of Antinephrin Antibodies

Plasma samples were collected from the patients before renal biopsy and were subsequently stored at −80 °C until analysis, avoiding repeated freeze-thaw cycles. Circulating antinephrin antibodies, both IgG and IgM, were measured using enzyme-linked immunosorbent assay according to our previously published protocol.^8^ The cutoff value for antibody positivity was 100 RU/ml for antinephrin IgG and 80 RU/ml for antinephrin IgM.

### Definitions

Hypoalbuminemia was defined as serum albumin < 30 g/l. NRP was defined as proteinuria > 3.5 g/d. NS was characterized by NRP and hypoalbuminemia. Treatment responses were recorded as follows: CR was defined as urinary protein excretion < 0.3 g/d, serum albumin > 35 g/l, and stable kidney function. Partial remission was defined as urinary protein < 3.5 g/d with a > 50% reduction from baseline, and stable eGFR or a decline ≤ 30% relative to baseline. No response was defined as a reduction in proteinuria < 50% from baseline or an increase in proteinuria, with or without deterioration in kidney function. Relapse was defined as urinary protein > 3.5 g/d after a period of remission. Follow-up time was defined as the interval from biopsy to the occurrence of an end point, or to the last visit for censored patients. The composite renal end point consisted of end-stage kidney disease—defined as eGFR < 15 ml/min per 1.73 m^2^ or initiation of kidney replacement therapy (dialysis or transplantation)—or a > 50% decline in eGFR from baseline.

### Statistical Analyses

Statistical analysis was performed using SPSS 26.0 (IBM, New York, NY). Categorical variables were presented as frequency or percentage (%). Continuous variables were described as mean ± SD for variables with a normal distribution or median and interquartile range for variables with a nonnormal distribution. Comparisons were performed using the *t* test or 1-way analysis of variance for variables with a normal distribution, or the Kruskal-Wallis test or Mann-Whitney U test for variables with a nonnormal distribution. Differences in categorical variables were assessed using the chi-square test or Fisher exact test. Spearman’s rank correlation coefficients were used to assess the relationships between antinephrin antibody levels and various clinical parameters. Multivariable logistic regression analyses were performed to identify independent predictors of CR in the overall cohort of patients with IgAN with NRP and in the subgroup of patients with NS. The models included antinephrin status, presence of MCD lesion, baseline proteinuria, eGFR, Oxford classification scores (M, E, S, T, C), and corticosteroid use. Odds ratios (ORs) and 95% confidence intervals (CIs) were reported. All analyses were 2-tailed, and *P* < 0.05 was considered statistically significant.

## Results

### Positive Rates of Antinephrin Antibodies

**Figure 1:**
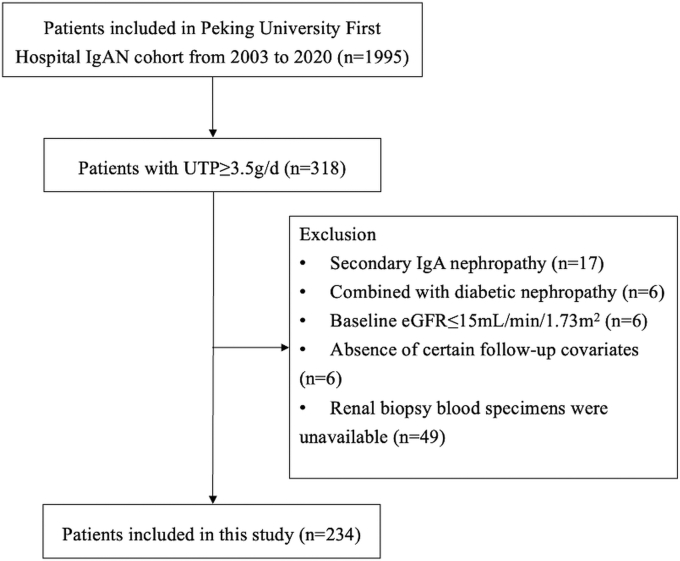
The flow chart for patients’ selection. eGFR, estimated glomerular filtration rate; IgAN, IgA nephropathy; UTP, urinary total protein.

**Figure 2:**
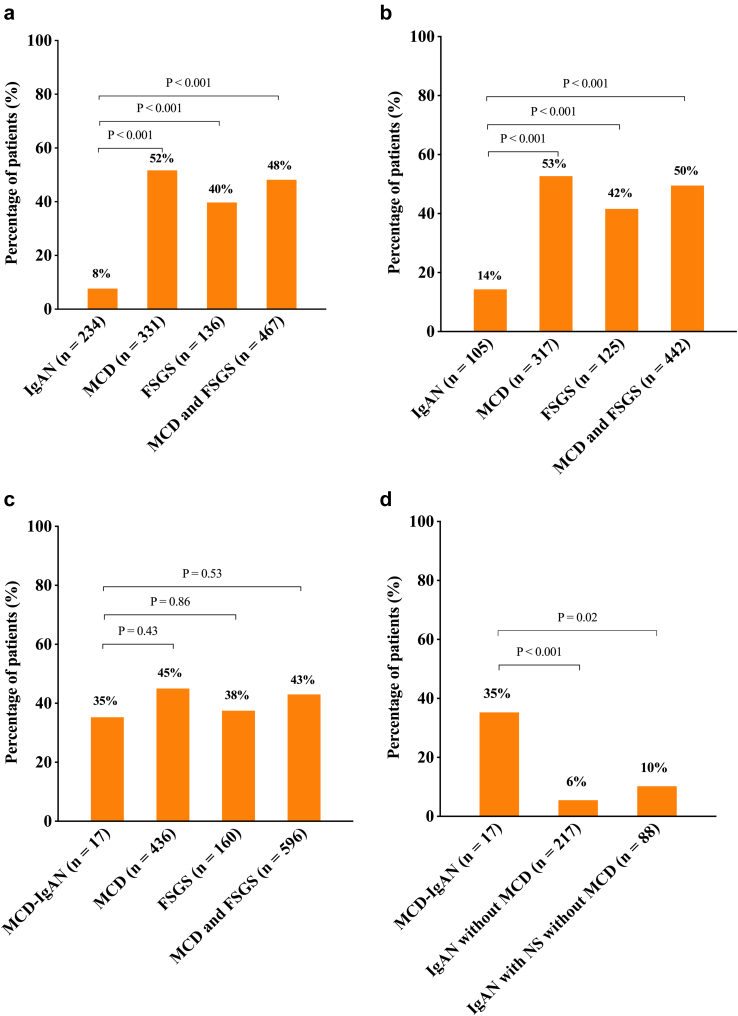
Positive rates of antinephrin antibodies in patients with different glomerular diseases. (a) Patients with nephrotic-range proteinuria (≥ 3.5 g/24 h): IgAN, MCD, primary FSGS, and MCD and primary FSGS cohorts combined. (b) Patients with nephrotic syndrome: IgAN, MCD, primary FSGS, and MCD and primary FSGS cohorts combined. (c) IgAN with MCD (MCD-IgAN) versus MCD, primary FSGS, and MCD and primary FSGS cohorts combined. (d) MCD-IgAN versus IgAN without MCD (overall and NS subset). FSGS, focal segmental glomerulosclerosis; IgAN, IgA nephropathy; MCD, minimal change disease; NS, nephrotic syndrome.

In Figure 1, we present the flow diagram of patient selection and exclusion criteria. A total of 234 patients with IgAN with NRP were included, among whom 105 had NS and 17 had MCD. We further compared the seropositivity of antinephrin antibodies with contemporaneous MCD and FSGS cohorts from our center (Figure 2); notably, the antinephrin antibody positive rates of these MCD and FSGS cohorts have been reported in previous studies.^8^

**Figure 3:**
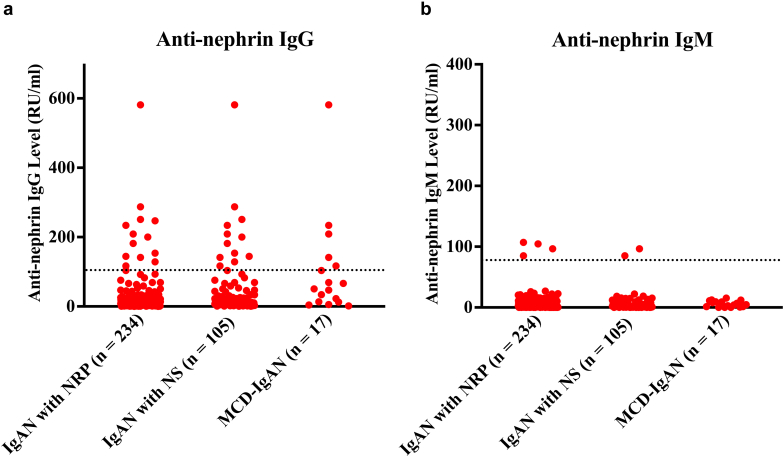
Serum antinephrin antibody levels in patients with different forms of IgA nephropathy. (a) Antinephrin IgG levels in patients with IgAN with nephrotic-range proteinuria (NRP), nephrotic syndrome (NS), and MCD-IgAN. (b) Antinephrin IgM levels in patients with IgAN with NRP, NS, and MCD-IgAN. IgAN, IgA nephropathy; MCD, minimal change disease.

Of the 234 patients with IgAN with NRP, 8% (18/234) tested positive for antinephrin antibodies—significantly lower than that in contemporaneous cohorts with NRP due to MCD or FSGS (8% vs. 52% and 40%, respectively; both P < 0.001; Figure 2a). The distribution of antibody levels in IgAN is further visualized in Figure 3: most patients across all 3 subgroups had antinephrin IgG and IgM levels below the positivity cutoff, with only a small proportion showing elevated levels (Figure 3a and b). Within the IgAN cohort, the positive rate of antinephrin IgG was 6% (14/234) and that of antinephrin IgM was 2% (4/234), with no double-positive cases for IgG and IgM.

In patients with IgAN presenting with NS, the positive rate of antinephrin antibodies was 14% (15/105), significantly lower than that in MCD (53%) and FSGS (42%) (both *P* < 0.001; Figure 2b). By contrast, in the subset of 17 patients with IgAN with coexisting MCD, the seropositivity of antinephrin antibodies reached 35% (6/17)—comparable to MCD (45%; *P* = 0.43) and FSGS (38%; *P* = 0.86; Figure 2c)—and markedly higher than in IgAN without coexisting MCD (6%, 12/217; *P* < 0.001). Among these 217 patients, those presenting with NS (a subset of 88 patients) showed a positivity rate of 10% (9/88), which remained significantly lower than MCD-IgAN (*P* = 0.02; Figure 2d).

### Antinephrin Antibodies and Clinical Characteristics

**Figure 4:**
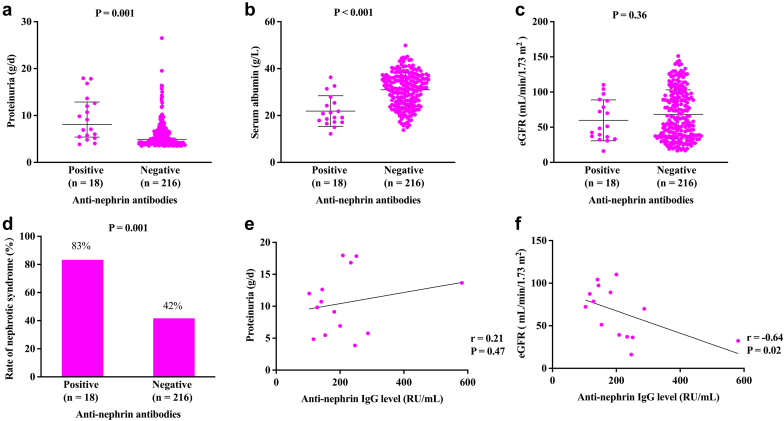
Clinical characteristics of IgAN with nephrotic-range proteinuria stratified by anti-nephrin antibody status. (a) 24-hour proteinuria; (b) serum albumin; (c) eGFR; (d) prevalence of nephrotic syndrome; (e and f) correlation plots of antinephrin IgG levels (RU/ml) with (e) proteinuria and (f) eGFR. eGFR, estimated glomerular filtration rate.

As shown in Figure 4 and Table 1, in the IgAN cohort with NRP, antinephrin antibody-positive patients had higher 24-hour urinary protein excretion and a higher prevalence of NS than antibody-negative patients (8.1 [5.4–12.9] vs. 4.9 [4.1–6.6] g/24 h, *P* = 0.001; 83% vs. 42%, P = 0.001). In contrast, positive patients had lower serum albumin (21.9 ± 6.5 vs. 31.0 ± 7.2 g/l, *P* < 0.001) and fewer urinary RBCs (3.0 [1.0–13.3] vs. 17.5 [4.3–67.5] RBCs/HPF, *P* = 0.002). No significant difference was observed in eGFR between the 2 groups (59.8 ± 29.1 vs. 68.3 ± 34.8 ml/min per 1.73 m^2^, *P* = 0.36). For patients positive for antinephrin IgG, antibody levels showed a weak nonsignificant positive correlation with 24-hour proteinuria (*r* = 0.21, *P* = 0.47) and a moderate inverse correlation with eGFR (*r* = −0.64, *P* = 0.02). Because of the extremely small sample size of antinephrin IgM-positive patients (*n* = 4), meaningful correlation analysis was not feasible; thus IgM scatterplots are not presented. On histopathology (Oxford classification), the antinephrin antibody-positive group had higher proportions of M0, S0, and T0 lesions (all *P* < 0.05), whereas no significant differences were noted in the E or C components (Table 1).

**Table 1:** Clinical characteristics of patients with IgAN with nephrotic-range proteinuria by antinephrin antibody status

Variables	Antinephrin antibodies		*P*-value
	Positive (*n* = 18)	Negative (*n* = 216)	
Male, *n* (%)	12 (67)	124 (57)	0.44
Age, yrs	40.3 ± 18.2	36.7 ± 15.0	0.48
MAP, mm Hg	99.7 ± 16.3	98.3 ± 10.9	0.61
BMI, kg/m^2^	26.8 ± 6.1	25.7 ± 4.1	0.67
NS, *n* (%)	15 (83)	90 (42)	0.001
ALB, g/l	21.9 ± 6.5	31.0 ± 7.2	< 0.001
Proteinuria, g/d	8.1 (5.4–12.9)	4.9 (4.1–6.6)	0.001
eGFR, ml/min per 1.73 m^2^	59.8 ± 29.1	68.3 ± 34.8	0.36
Hematuria, RBCs/HPF	3.0 (1.0–13.3)	17.5 (4.3–67.5)	0.002
Oxford classification			
M0/1	9/9	57/159	0.03
E0/1	7/11	117/99	0.21
S0/1	13/5	86/130	0.007
T0/1/2	13/4/1	76/86/54	0.007
C0/1/2	10/4/4	69/90/57	0.11
No treatment on biopsy, *n* (%)	18 (100)	208 (96)	1.00
Treatments, *n* (%)	*n* = 18	*n* = 216	
RAASi	16 (89)	195 (90)	0.69
Corticosteroids	18 (100)	161 (75)	0.009
Cyclophosphamides	4 (22)	68 (32)	0.41
Leflunomide	6 (33)	26 (12)	0.03
Mycophenolate mofetil	5 (28)	25 (12)	0.06
Calcineurin inhibitors	6 (33)	33 (15)	0.09
Responses to treatment, *n* (%)			0.006
Complete remission	12 (67)	64 (30)	
Partial remission	5 (28)	128 (60)	
No remission	1 (6)	24 (11)	
Relapse	7/17 (41)	65/192 (34)	0.54
Proteinuria remission duration, mos			
CR duration	5.8 (1.9–7.8)	8.8 (2.1–14.5)	0.15
PR duration	4.3 (2.2–8.6)	4.0 (2.1–6.8)	0.63
50% eGFR reduction, *n* (%)	0 (0)	56 (26)	0.008
ESKD, *n* (%)	0 (0)	27 (13)	0.24
Composite kidney end point, *n* (%)	0 (0)	60 (28)	0.009
Follow-up time, mos	29.7 (19.7–41.8)	38.7 (23.8–69.8)	0.11

ALB, serum albumin; BMI, body mass index; CR, complete remission; eGFR, estimated glomerular filtration rate; ESKD, end stage kidney disease; MAP, mean arterial pressure; NS, nephrotic syndrome; PR, partial remission; RAASi, renin-angiotensin-aldosterone system inhibitors; RBCs, red blood cells.

Continuous variables were expressed as mean ± SD or median (interquartile range). Categorical variables were expressed as number (%).

Composite kidney end point was defined as the first occurrence of a sustained 50% decrease in eGFR or ESKD.

In the NS subgroup (Supplementary Table S1), antinephrin-positive patients consistently showed heavier proteinuria, lower serum albumin, fewer urinary RBCs, and higher proportions of M0 and T0 lesions (all *P* < 0.05), with no significant differences in other clinical indices or the E/S/C components of the Oxford classification.

In IgAN with coexisting MCD (Supplementary Table S2), NS was universal (100%), compared with 41% in patients without coexisting lesions (*P* < 0.001). These patients had heavier proteinuria (10.0 [5.7–13.2] vs. 4.9 [4.1–6.6] g/d, *P* < 0.001), lower serum albumin (20.5 ± 4.3 vs. 31.0 ± 7.2 g/l, *P* < 0.001), higher eGFR (91.1 ± 34.3 vs. 65.8 ± 33.8 ml/min per 1.73 m^2^, *P* = 0.007), and fewer urinary RBCs (3.0 [1.3–18.8] vs. 17.5 [4.0–65.0] RBCs/HPF, *P* = 0.006). Correspondingly, the seropositivity of antinephrin antibodies was markedly higher in the coexisting group (35% vs. 6%, *P* < 0.001), which was driven by antinephrin IgG (35% vs. 4%, *P* < 0.001; antinephrin IgM: 0% vs. 2%, *P* = 1.000). Antinephrin IgG levels were higher in the coexisting group (50.8 [12.9–129.1] vs. 9.3 [4.6–18.4] RU/ml, *P* < 0.001). Histopathologically, the coexisting group had milder lesions overall, with more M0/S0/T0 lesions, fewer T1/T2 lesions (all *P* ≤ 0.009), and reduced proportions of E1 and C1/C2 lesions (*P* = 0.04 and *P* = 0.003, respectively).

In antinephrin-positive patients with IgAN (Supplementary Table S3), those with coexisting MCD and those without were clinically similar; proteinuria, serum albumin, eGFR, and hematuria did not differ significantly. Antinephrin IgG levels were comparable (175.0 [113.5–320.7] vs. 148.9 [136.6–235.2] RU/ml; *P* = 0.55), as were IgM levels (*P* = 0.25). By contrast, histopathology was milder with coexisting MCD.

### Antinephrin Antibodies and Treatment Responsiveness and Renal Outcomes

In IgAN with NRP (Table 1), antinephrin-positive patients were more likely to receive glucocorticoids (100% vs. 75%, *P* = 0.009) and leflunomide (33% vs 12%, *P* = 0.03) than antibody-negative patients, whereas no significant differences were observed in the usage of other immunosuppressive agents. In terms of treatment responsiveness, the CR rate was higher in the antinephrin-positive group (67% vs. 30%, *P* = 0.006), whereas relapse rate and duration of remission were similar. Regarding renal outcomes, antinephrin-positive patients had fewer events of eGFR decline ≥ 50% and fewer composite renal end points (0% vs. 26%, *P* = 0.008; 0% vs. 28%, *P* = 0.009).

In the NS subgroup (Supplementary Table S1), the usage of medications was broadly similar between antinephrin-positive and -negative patients. The positive group showed a higher CR rate (80% vs. 43%, *P* = 0.03), with similar relapse rates. The rates of eGFR decline ≥ 50% and the composite end points were 0% in the positive group versus 23% in the negative group (*P* = 0.04).

Multivariable logistic regression analysis was performed to identify independent predictors of CR. In IgAN with NRP, glomerular sclerosis (S1 vs. S0, OR = 0.24, 95% CI: 0.11–0.52, *P* < 0.001), crescent formation (C2 vs. C0, OR = 0.25, 95% CI: 0.08–0.75, *P* = 0.01), and corticosteroid use (OR = 3.23, 95% CI: 1.30–8.00, *P* = 0.01) were significant independent predictors. Antinephrin positivity showed a trend toward higher CR rates (OR = 3.44, 95% CI: 0.87–13.63, *P* = 0.08). Similar trends were observed in the NS subgroup (Supplementary Table S4 and S5).

Comparing IgAN with versus without coexisting MCD (Supplementary Table S2), the coexisting group achieved a higher CR rate (71% vs. 30%, *P* = 0.002); relapse did not differ. Events of eGFR decline ≥ 50% and the composite end points were numerically lower in the coexisting group (both 6% vs. 25% and 27%, respectively), but were not statistically significant.

Within the antinephrin-positive subset (Supplementary Table S3), the subgroup with coexisting MCD achieved a higher CR rate (100% vs. 50%, *P* = 0.04) without excess adverse renal events; no cases of eGFR decline ≥ 50%, end-stage kidney disease, or the composite end points occurred in either group (all 0%). Of note, these subgroup comparisons are based on small sample sizes and statistically underpowered; these results should therefore be interpreted with caution and regarded as exploratory findings.

## Discussion

In this study, we systematically investigated the prevalence and clinical relevance of circulating antinephrin autoantibodies in a large adult cohort with biopsy-proven IgAN and NRP and NS, a population insufficiently assessed in previous studies that predominantly examined MCD and primary FSGS.^8^^,^^9^

In our cohort, the antinephrin antibodies positivity among patients with IgAN presenting with NS was 14%, markedly lower than in NS attributable to MCD or primary FSGS. Historically, NS in IgAN was often equated with superimposed MCD, and corticosteroids were commonly prescribed on the assumption of MCD-like steroid responsiveness.^12^^,^^13^ However, subsequent studies indicate that nephrotic IgAN encompasses ≥2 distinct histopathologic patterns: a typical form with endocapillary hypercellularity and crescents on light microscopy and marked foot-process effacement on electron microscopy, and an MCD-like form characterized by only mild mesangial change on light microscopy but diffuse effacement consistent with podocytopathy.^14^^,^^15^ This disparity in antinephrin positivity between NS-IgAN and MCD and FSGS further supports the heterogeneity of nephrotic IgAN and suggests that not all cases are driven by immune podocytopathy. Importantly, seropositivity was observed in IgAN without MCD, indicating that nephrin autoimmunity is not restricted to overt podocytopathy and may arise in a broader subset of IgAN with nephrotic presentations. Notably, such seropositivity in patients without histologically diagnosed podocytopathy may be explained in part by misclassification of subtle podocytopathy. Standard electron microscopy criteria for podocytopathy rely on diffuse foot-process effacement, whereas mild or focal podocyte injury may be underrecognized and classified as negative for podocytopathy. Biopsy timing may contribute, because podocyte injury could be in an early or resolving phase without fully developed morphological changes. Therefore, seropositivity in this subgroup likely reflects unrecognized mild podocyte injury rather than antibody occurrence in the absence of podocytopathy.

In the subset with biopsy-proven IgAN with MCD, the prevalence of circulating antinephrin antibodies was 35%, lower than reported in pediatric IgAN-MCD,^16^ but comparable to the positivity observed in our adult MCD and primary FSGS cohorts,^8^ and significantly higher than in classic IgAN without MCD. Together with prior evidence that antinephrin antibodies disrupt the slit diaphragm, induce foot-process effacement, and are associated with podocyte injury in MCD and FSGS,^9^^,^^17^ these observations suggest that podocyte injury in the IgAN-MCD subgroup may be associated with antinephrin antibodies and distinct from the immune-complex–driven mesangial and endocapillary injury typical of classic IgAN.^18^

In IgAN with NRP or NS, antinephrin-positive patients consistently exhibited heavier proteinuria, lower serum albumin, and less hematuria; moreover, clinical severity correlated with antibody levels, consistent with prior observations in MCD and FSGS.^8^ Histologically, Oxford classification lesions were predominantly M0/S0/T0, with no enrichment of E or C components, consistent with low glomerular activity and minimal mesangial and endocapillary proliferation, arguing against an inflammatory glomerulonephritis-driven proteinuria. This clinicopathologic constellation is more compatible with a podocytopathy-like phenotype—characterized by slit-diaphragm dysfunction and foot-process effacement with relatively limited glomerular inflammatory activity.^7^^,^^19^ These findings further support the presence of ≥2 pathogenic pathways within IgAN-NS.^14^

Regarding treatment response and outcomes, antinephrin-positive patients with NRP achieved higher CR rates and experienced fewer renal endpoint events during follow-up; similar trends were observed in the NS subgroup. Consistent with these findings, multivariable logistic regression analysis showed that antinephrin positivity was associated with a trend toward higher CR rates, though it did not reach formal statistical significance. Patients with MCD-IgAN likewise showed higher CR rates. These findings suggest that antinephrin testing may serve as a stratification tool to identify a steroid-responsive, podocytopathy-leaning phenotype within NS-IgAN. Nevertheless, seropositive patients in our cohort were more likely to receive glucocorticoids, introducing potential treatment-selection bias, and posttreatment antibody levels were not assessed. Therefore, the association between antinephrin status and therapeutic response should be confirmed in prospective, multicenter studies with standardized protocols and longitudinal serologic monitoring.

In accordance with the Kidney Disease: Improving Global Outcomes guidelines, the treatment approach for patients with IgAN-NS is contingent upon pathological findings.^20^ For example, kidney biopsies demonstrating mesangial IgA deposition along with light and electron microscopy features consistent with MCD should be managed similarly to MCD. Building on our findings, adding antinephrin antibodies testing as an adjunct to renal biopsy may further refine risk stratification in IgAN-NS by identifying a podocytopathy-leaning, steroid-responsive subset in whom earlier induction and closer relapse surveillance are warranted, whereas seronegativity—particularly with prominent endocapillary activity or chronic scarring—supports management pathways used for inflammation-dominant, high-risk IgAN. This approach would be a valuable complement to current guideline-based algorithms. For clinical implementation, targeted antinephrin testing may be performed in patients with IgAN with NRP or NS, especially those with minimal hematuria and an M0/S0/T0 phenotype. A positive result could support earlier corticosteroid treatment and closer monitoring for relapse. However, false-positive results may lead to unnecessary immunosuppression. Currently, no standardized commercial assay is available, and our findings are limited to a single Chinese center. Multicenter validation is thus needed before broad clinical use.

Two recent studies have explored antinephrin antibodies in IgAN with NS. Ying *et al.*^16^ focused on a pediatric cohort and reported high antinephrin positivity in IgAN-MCD but no positivity in typical proliferative IgAN-NS. Yang *et al.*^21^ identified an IgAN-MCD overlap phenotype using renal tissue IgG-nephrin colocalization, but did not measure circulating antinephrin antibodies in serum. The present study extends these observations by investigating circulating antinephrin antibodies in a large adult cohort with long-term follow-up. We demonstrate that antinephrin seropositivity enriches for a podocytopathy-like phenotype not only in IgAN with histologically confirmed MCD, but also in a subset of adult NS-IgAN without overt podocytopathy. In addition, we provide long-term renal outcome data showing favorable prognosis in seropositive patients, which has not been reported previously. Our findings therefore expand the potential utility of antinephrin antibodies from pediatric populations and histologically defined MCD cases to a broader adult population with IgAN and NRP.

Our study has several limitations that should be acknowledged. First, this was a single-center, retrospective analysis with single time-point serology. Most importantly, the overall number of antinephrin-positive patients was small (*n* = 18), and key subgroups were even smaller, which limited statistical power and generalizability of subgroup comparisons. Second, we only evaluated antinephrin antibodies and did not investigate other recently identified pathogenic antislit diaphragm autoantibodies, including antipodocin and anti-Kirrel1, which may also contribute to podocyte injury in patients with IgAN-NS.^22^ In addition, we did not assess posttreatment antibody dynamics, perform tissue-level immunofluorescence or colocalization of antinephrin antibodies, or profile other podocyte-related autoantibodies and complement in parallel. Because of the retrospective design and limited residual renal biopsy specimens, we were unable to further verify intrarenal antibody deposition, which limits mechanistic resolution and precludes confirmation of a direct pathogenic role. In addition, 49 patients were excluded due to lack of available serum samples, which may introduce a slight risk of selection bias. Finally, small differences in follow-up duration may affect event accrual, and the generalizability of our findings across populations and assay platforms remains to be established.

In conclusion, antinephrin antibodies are uncommon in adult IgAN but enriched in cases with coexisting MCD, defining a podocytopathy-leaning phenotype. Notably, seropositivity occurred in IgAN without these lesions, indicating that nephrin autoimmunity extends beyond overt podocytopathy and is associated with a broader subset of nephrotic IgAN. Antinephrin testing may refine phenotypic stratification and guide monitoring or treatment decisions, a possibility that warrants prospective validation.

## Disclosure

All the authors declared no competing interests.

## Supplementary Material

Supplementary File (PDF) **Table S1.** Clinical characteristics of IgAN patients with nephrotic syndrome by antinephrin antibody status. **Table S2.** Clinical characteristics of IgAN with and without coexisting MCD. **Table S3.** Clinical characteristics of antinephrin-positive IgAN with and without coexisting MCD. **Table S4.** Multivariable logistic regression for independent predictors of complete remission in IgAN patients with NRP. **Table S5.** Multivariable logistic regression for independent predictors of complete remission in IgAN patients with nephrotic syndrome.

## Footnotes

Supplementary File (PDF)
**Table S1.** Clinical characteristics of IgAN patients with nephrotic syndrome by antinephrin antibody status. **Table S2.** Clinical characteristics of IgAN with and without coexisting MCD. **Table S3.** Clinical characteristics of antinephrin-positive IgAN with and without coexisting MCD. **Table S4.** Multivariable logistic regression for independent predictors of complete remission in IgAN patients with NRP. **Table S5.** Multivariable logistic regression for independent predictors of complete remission in IgAN patients with nephrotic syndrome.
