# Supplementary material for: Circulating Antinephrin Antibodies in Adult Chinese Patients With IgAN and Nephrotic-Range Proteinuria
Source: Kidney Int Rep. 2026 Jun 2;11(8):106640. doi: 10.1016/j.ekir.2026.106640 (PMC13329496; doi:10.1016/j.ekir.2026.106640)
Supplement: Supplementary File (PDF) — Table S1. Clinical characteristics of IgAN patients with nephrotic syndrome by antinephrin antibody status. Table S2. Clinical characteristics of IgAN with and without coexisting MCD. Table S3. Clinical characteristics of antinephrin-positive IgAN with and without coexisting MCD. Table S4. Multivariable logistic regression for independent predictors of complete remission in IgAN patients with NRP. Table S5. Multivariable logistic regression for independent predictors of complete remission in IgAN patients with nephrotic syndrome. [file mmc1.pdf]

Supplementary Material

Table S1. Clinical characteristics of IgAN patients with nephrotic syndrome by anti-nephrin antibody status

Table S2. Clinical characteristics of IgAN with and without coexisting MCD

Table S3. Clinical characteristics of anti-nephrin–positive IgAN with and without coexisting MCD

Table S4. Multivariable logistic regression for independent predictors of complete remission in IgAN patients with nephrotic-range proteinuria

Table S5. Multivariable logistic regression for independent predictors of complete remission in IgAN patients with nephrotic syndrome

Table S1. Clinical characteristics of IgAN patients with nephrotic syndrome by anti-nephrin antibody status.

	Anti-nephrin antibodies		P value
	Positive (n = 15)	Negative (n = 90)	
Male, n (%)	10 (67)	48 (53)	0.34
Age, years	39.8 ± 17.9	36.4 ± 17.5	0.48
MAP, mmHg	97.7 ± 15.3	96.2 ± 10.4	0.59
BMI, kg/m^2	27.1 ± 6.4	24.5 ± 3.9	0.10
ALB, g/L	19.6 ± 4.1	23.9 ± 4.0	**0.001**
Proteinuria, g/d	9.8 (5.5-13.7)	6.0 (4.3-8.2)	**0.01**
eGFR, mL/min/1.73 m^2	65.8 ± 27.8	80.4 ± 36.1	0.13
Hematuria, RBCs/HPF	2.3 (1.0-7.1)	28.8 (6.6-106.3)	**< 0.001**
Oxford classification			
M0/1	9/6	30/60	**0.048**
E0/1	7/8	48/42	0.63
S0/1	12/3	53/37	0.12
T0/1/2	13/2/0	41/30/19	**0.009**
C0/1/2	10/3/2	35/32/23	0.13
No treatment on biopsy, n (%)	15 (100)	84 (93)	0.59
Treatments, n (%)	n = 15	n = 90	
RAASi	14 (93)	79 (88)	0.85
Corticosteroids	15 (100)	78 (87)	0.21
Cyclophosphamides	3 (20)	30 (33)	0.38
Leflunomide	5 (33)	11 (12)	0.05
Mycophenolate mofetil	2 (13)	9 (10)	0.66
Calcineurin inhibitors	6 (40)	23 (26)	0.35
Responses to treatment, n (%)			**0.03**
Complete remission	12 (80)	39 (43)	
Partial remission	2 (13)	42 (47)	
No remission	1 (7)	9 (10)	
Relapse	6/14 (43)	29/81 (36)	0.61
Proteinuria remission duration, months			
CR duration	5.8 (1.9-7.8)	5.6 (1.1-12.2)	0.89
PR duration	6.3 (3.7-8.9)	4.6 (2.5-7.3)	0.51
50% eGFR reduction, n (%)	0 (0)	21 (23)	**0.04**
ESKD, n (%)	0 (0)	6 (7)	0.59
Composite kidney endpoint, n (%)	0 (0)	21 (23)	**0.04**
Follow-up time, months	30.2 (17.7-41.0)	41.0 (26.5-67.5)	0.12

Continuous variables were expressed as mean ± standard deviation or median (25th percentile-75th percentile). Categorical variables were expressed as number (percent). MAP, mean arterial pressure; BMI, body mass index; NS, nephrotic syndrome; ALB, serum albumin; eGFR, estimated glomerular filtration rate; RAASi, renin–angiotensin–aldosterone system inhibitors; CR, complete remission;

PR, partial remission; ESKD, end stage kidney disease; Composite kidney endpoint was defined as the first occurrence of a sustained 50% decreases in eGFR or ESKD.

Table S2. Clinical characteristics of IgAN with and without coexisting MCD.

	IgAN with MCD (n = 17)	IgAN without MCD (n = 217)	P value
Male, n (%)	12 (71)	124 (57)	0.28
Age, years	42.3 ± 21.9	36.5 ± 14.6	0.53
MAP, mmHg	97.3 ± 12.6	98.4 ± 11.3	0.50
BMI, kg/m2	26.5 ± 4.8	25.8 ± 4.3	0.37
NS, n (%)	17 (100)	88 (41)	**< 0.001**
ALB, g/L	20.5 ± 4.3	31.0 ± 7.2	**< 0.001**
Proteinuria, g/d	10.0 (5.7-13.2)	4.9 (4.1-6.6)	**< 0.001**
eGFR, mL/min/1.73 m2	91.1 ± 34.3	65.8 ± 33.8	**0.007**
Hematuria, RBCs/HPF	3.0 (1.3-18.8)	17.5 (4.0-65.0)	**0.006**
Anti-nephrin antibody levels, RU/mL			
Anti-nephrin IgG	50.8 (12.9-129.1)	9.3 (4.6-18.4)	**< 0.001**
Anti-nephrin IgM	4.7 (1.0-10.2)	2.5 (1.0-6.5)	0.40
The positive rates of anti-nephrin antibodies, n (%)	6 (35)	12 (6)	**< 0.001**
Anti-nephrin IgG	6 (35)	8 (4)	**< 0.001**
Anti-nephrin IgM	0 (0)	4 (2)	1.00
Oxford classification			
M0/1	13/4	53/164	**< 0.001**
E0/1	13/4	111/106	**0.04**
S0/1	15/2	84/133	**< 0.001**
T0/1/2	15/2/0	74/88/55	**< 0.001**
C0/1/2	12/4/1	67/90/60	**0.003**
No treatment on biopsy, n (%)	17 (100)	209 (96)	1.00
Treatments, n (%)	n = 17	n = 217	
RAASi	11 (65)	200 (92)	**0.003**
Corticosteroids	15 (88)	164 (76)	0.37
Cyclophosphamides	2 (12)	70 (32)	0.08
Leflunomide	4 (24)	28 (13)	0.26
Mycophenolate mofetil	0 (0)	30 (14)	0.14
Calcineurin inhibitors	5 (29)	34 (16)	0.17
Responses to treatment, n (%)			**0.002**
Complete remission	12 (71)	64 (30)	
Partial remission	5 (29)	128 (59)	
No remission	0 (0)	25 (12)	
Relapse	3/17 (18)	68/192 (35)	0.14
Proteinuria remission duration, months			
CR duration	5.9 (0.7-9.7)	8.3 (2.2-14.3)	0.13
PR duration	2.9 (2.1-4.7)	4.1 (2.1-6.8)	0.84
50% eGFR reduction, n (%)	1 (6)	55 (25)	0.08

ESKD, n (%)	0 (0)	27 (12)	0.23
Composite kidney endpoint, n (%)	1 (6)	59 (27)	0.08
Follow-up time, months	34.2 (20.3-43.2)	38.5 (23.7-71.8)	0.17

Continuous variables were expressed as mean ± standard deviation or median (25th percentile-75th percentile). Categorical variables were expressed as number (percent). MAP, mean arterial pressure; BMI, body mass index; NS, nephrotic syndrome; ALB, serum albumin; eGFR, estimated glomerular filtration rate; RAASi, renin–angiotensin–aldosterone system inhibitors; CR, complete remission; PR, partial remission; ESKD, end stage kidney disease; Composite kidney endpoint was defined as the first occurrence of a sustained 50% decreases in eGFR or ESKD.

Table S3. Clinical characteristics of anti-nephrin-positive IgAN with and without coexisting MCD.

	IgAN with MCD (n = 6)	IgAN without MCD (n = 12)	P value
Male, n (%)	5 (83)	7 (58)	0.60
Age, years	44.1 ± 19.8	38.3 ± 18.0	0.68
MAP, mmHg	93.1 ± 13.3	102.9 ± 17.1	0.18
BMI, kg/m^2	26.8 ± 6.2	26.9 ± 6.4	0.82
NS, n (%)	6 (100)	9 (75)	0.52
ALB, g/L	18.8 ± 4.4	23.4 ± 7.0	0.29
Proteinuria, g/d	12.8 (9.2-17.1)	6.5 (5.2-9.6)	0.07
eGFR, mL/min/1.73 m^2	62.1 ± 30.2	58.7 ± 29.8	0.89
Hematuria, RBC/HPF	1.5 (0.75-3.0)	6.5 (1.0-35.0)	0.06
Anti-nephrin antibody levels, RU/mL			
Anti-nephrin IgG	175.0 (113.5-320.7)	148.9 (13.6-235.2)	0.55
Anti-nephrin IgM	1.8 (0.1-8.3)	2.6 (1.2-93.7)	0.18
The positive rates of anti-nephrin antibodies, n (%)			
Anti-nephrin IgG	6 (100)	8 (67)	0.25
Anti-nephrin IgM	0 (0)	4 (33)	0.25
Oxford classification			
M0/1	5/1	4/8	0.13
E0/1	6/0	1/11	**< 0.001**
S0/1	6/0	7/5	0.11
T0/1/2	6/0/0	7/4/1	0.08
C0/1/2	6/0/0	4/4/4	**0.009**
No treatment on biopsy, n (%)	6 (100)	12 (100)	-
Treatments, n (%)	n = 6	n = 12	
RAASi	5 (83)	11 (92)	1.00
Corticosteroids	6 (100)	12 (100)	-
Cyclophosphamides	1 (17)	3 (25)	1.00
Leflunomide	1 (17)	5 (42)	0.60
Mycophenolate mofetil	0 (0)	5 (42)	0.11
Calcineurin inhibitors	3 (50)	3 (25)	0.34
Responses to treatment, n (%)			**0.04**
Complete remission	6 (100)	6 (50)	
Partial remission	0 (0)	5 (42)	
No remission	0 (0)	1 (8)	
Relapse	1/6 (17)	5/11 (46)	0.33
Proteinuria remission duration, months			
CR duration	6.4 (4.4-8.6)	3.2 (1.6-7.6)	0.49
PR duration	-	4.3 (2.2-8.6)	-

50% eGFR reduction, n (%)	0 (0)	0 (0)	-
ESKD, n (%)	0 (0)	0 (0)	-
Composite kidney endpoint, n (%)	0 (0)	0 (0)	-
Follow-up time, months	27.9 (16.8-47.9)	30.0 (20.9-42.9)	0.75

Continuous variables were expressed as mean ± standard deviation or median (25th percentile-75th percentile). Categorical variables were expressed as number (percent). MAP, mean arterial pressure; BMI, body mass index; NS, nephrotic syndrome; ALB, serum albumin; eGFR, estimated glomerular filtration rate; RAASi, renin–angiotensin–aldosterone system inhibitors; CR, complete remission; PR, partial remission; ESKD, end stage kidney disease; Composite kidney endpoint was defined as the first occurrence of a sustained 50% decreases in eGFR or ESKD.

Table S4. Multivariable logistic regression for independent predictors of complete remission in IgAN patients with nephrotic-range proteinuria.

Variable	OR (95% CI)	P value
Anti-nephrin positive	3.44 (0.87-13.63)	0.08
Presence of MCD lesion	0.84 (0.21-3.38)	0.81
Proteinuria, g/d	1.04 (0.92-1.17)	0.54
eGFR, mL/min/1.73 m^2	1.01 (1.00-1.02)	0.05
Oxford classification		
M1	0.49 (0.21-1.15)	0.10
E1	0.52 (0.24-1.12)	0.10
S1	0.24 (0.11-0.52)	<0.001
T1	1.60 (0.62-4.13)	0.34
T2	0.33 (0.07-1.53)	0.16
C1	1.14 (0.47-2.73)	0.78
C2	0.25 (0.08-0.75)	0.01
Steroid use	3.23 (1.30-8.00)	0.01

OR, odds ratio; CI, confidence interval

Table S5. Multivariable logistic regression for independent predictors of complete remission in IgAN patients with nephrotic syndrome.

Variable	OR (95% CI)	P value
Anti-nephrin positive	4.56 (0.75-27.69)	0.10
Presence of MCD lesion	0.35 (0.07-1.81)	0.21
Proteinuria, g/d	1.11 (0.93-1.31)	0.25
eGFR, mL/min/1.73 m^2	1.02 (1.00-1.04)	0.11
Oxford classification		
M1	0.73 (0.18-2.88)	0.65
E1	0.21 (0.06-0.81)	0.02
S1	0.29 (0.09-0.93)	0.04
T1	1.31 (0.31-5.46)	0.71
T2	0.07 (0.01-0.89)	0.04
C1	0.97 (0.22-4.33)	0.97
C2	0.16 (0.03-0.95)	0.04
Steroid use	1.28 (0.26-6.27)	0.76

OR, odds ratio; CI, confidence interval
